# Overview of Donkey Welfare and Husbandry Practices in Asia

**DOI:** 10.3390/ani15233464

**Published:** 2025-12-01

**Authors:** Abd Ullah, Muhammad Zahoor Khan, Changfa Wang

**Affiliations:** College of Agriculture and Biology, Liaocheng University, Liaocheng 252000, China

**Keywords:** donkey, welfare, Asia, veterinary care, management, exploitation, conservation

## Abstract

Donkeys (*Equus asinus*) play a critical role in agricultural, transport, and livelihood systems across Asia, yet they remain among the most neglected domestic species in terms of welfare, management, and research attention. This review synthesizes recent literature on donkey welfare, health, breeding, and conservation across Asia, highlighting regional disparities and emerging challenges. A systematic review of published studies identified welfare determinants including nutrition, workload, shelter, and veterinary access. Welfare conditions are found to be poorest in South Asia, particularly in Pakistan, India, and Afghanistan, where chronic undernutrition, inadequate housing, excessive workloads, and limited veterinary support prevail. Preventive healthcare, such as vaccination and deworming, remains largely absent, reflecting low owner awareness and weak veterinary infrastructure. In contrast, China demonstrates substantial progress through semi-intensive farming systems, structured welfare management, and research-based breeding programs that integrate welfare with productivity enhancement. Recent advancements in molecular genetics have further expanded the scope of donkey conservation and improvement. Studies on key genes, including *PRKG2*, *NR6A1*, *LTBP2*, *HOXC8*, and *DCAF7*, have elucidated their roles in vertebral number, skeletal development, and body conformation in Dezhou donkeys, offering new opportunities for genomic-level conservation and marker-assisted selection. Nonetheless, significant health challenges, such as parasitic, bacterial, and viral infections (*Theileria equi*, *Giardia duodenalis*, and *Equid herpesviruses*), continue to threaten productivity and welfare. Reproductive management across most Asian countries remains traditional and uncoordinated, whereas China leads in artificial insemination, genetic resource preservation, and policy-supported breeding initiatives. Ethical concerns surrounding overexploitation and the commercial use of donkeys, particularly in the ejiao (donkey-hide gelatin) industry, are also gaining attention. Overall, this review underscores the urgent need for a “One Welfare” approach, linking Animal Welfare, human livelihoods, and sustainable industry development. Strengthening veterinary infrastructure, promoting owner education, and integrating genomic tools into breeding programs are essential steps toward improving the welfare, productivity, and long-term conservation of donkeys across Asia.

## Simple Summary

This review examines the diverse welfare, health, and management conditions of donkeys across Asia. It highlights severe challenges in South Asia, including overwork and poor veterinary care, contrasting with China’s more structured, industry-driven welfare and breeding programs. The scope encompasses husbandry practices, disease management, conservation efforts, and ethical concerns, advocating for a “One Welfare” approach that integrates animal well-being with human livelihood and sustainable development.

## 1. Introduction

Donkeys (*Equus asinus*) have been indispensable companions to humanity for over 5000 years, playing crucial roles in Agriculture, transportation, and traditional livelihoods [1,2]. Despite their long history of service, they remain one of the most overlooked and undervalued species in global discussions on Animal Welfare [2,3]. According to FAOSTAT, the global donkey population was estimated to be approximately 50 million, with Africa accounting for the largest share at 61%. Asia follows with 26%, [2,4,5]. In China, millions of donkeys are integral to traditional farming practices. In contrast, in other regions of Asia, donkeys continue to play an essential role in the daily lives of rural communities [4,6]. Donkeys remain vital to rural communities across the continent, offering crucial services in Agriculture and transportation that mechanization has yet to replace [4]. From transporting goods in the mountainous regions of Central Asia to supporting smallholder farming in South Asia, donkeys are integral to local economies and sustainable agricultural practices [7].

In addition to their roles in transportation and Agriculture, donkeys are notably in demand for donkey-derived products, particularly in traditional Chinese medicine (ejiao), meat production, and milk harvesting [8,9,10,11]. Despite their fundamental importance to global food security, poverty alleviation, and rural development, donkeys face a constellation of interconnected challenges that threaten both their welfare and the communities that depend upon them [4,12]. These challenges span infectious diseases, nutritional deficiencies, physical abuse, and inadequate veterinary care, compounded by their low social status in many cultures and the frequent misconception that donkeys are naturally hardy and require minimal care [13,14,15,16]. Climate change is simultaneously intensifying the harsh environments in which many working donkeys operate, while urbanization and mechanization are altering traditional donkey-keeping practices without necessarily improving Animal Welfare outcomes [17,18].

Despite their historical and ongoing contributions, donkeys remain one of the most underappreciated and often neglected livestock species, especially in developing regions [1,19]. In Asia, the welfare and management of donkeys present unique challenges due to socio-economic factors, cultural attitudes, and limited access to veterinary services [20]. The welfare of donkeys in Asia is intricately linked to several key factors, including their health, nutrition, reproductive management, and the conditions under which they work [20,21]. While donkeys are relatively hardy Animals, they face a range of health issues, from parasitic infestations to injuries caused by overwork, which are exacerbated by insufficient care and inadequate husbandry practices [20,21,22]. Feeding practices and reproductive management also play critical roles in ensuring donkey welfare [23]. Despite their ability to graze on low-quality forage, donkeys require specific nutritional care to maintain health, productivity, and longevity [24]. However, inadequate feeding and poor reproductive practices can lead to a decline in donkey health, affecting their capacity to work and reproduce effectively [25]. Moreover, cultural perceptions of donkeys in many Asian countries influence their treatment, with some communities valuing them primarily for their utility rather than their welfare [20,26]. This utilitarian view, coupled with the lack of organized welfare standards, often results in suboptimal living conditions and labor demands that exceed their physiological limits.

Furthermore, the availability of vaccines and preventive medicine is often limited in remote areas, putting donkey populations at risk of infectious diseases [27,28,29]. The lack of organized healthcare, combined with limited access to veterinary services, worsens their health outcomes [30,31,32]. Previous reviews have largely focused on Africa, Europe, or working equids in general, and they often rely on outdated demographic data or do not incorporate recent advances in disease surveillance, reproductive management, and genetic improvement [33,34]. A recent review briefly explained the welfare and husbandry practices of donkeys in Europe [35]. No comprehensive or up-to-date publication has evaluated donkey welfare and management throughout the Asian continent, nor integrated recent advancements in disease surveillance, reproductive management, and genetic improvement from Asian countries. Therefore, this review aims to fill this critical gap by systematically evaluating the current state of donkey welfare, husbandry practices, and management systems throughout the Asian region. This review aims to provide a comprehensive overview of donkey welfare and husbandry practices in Asia, focusing on key aspects such as shelter and food, health, vaccination, nutrition, and reproduction. Through an in-depth examination of existing literature and current practices, this article seeks to highlight the challenges facing donkey husbandry in Asia and propose recommendations to improve their welfare. By improving the standards of care and providing better resources, the quality of life for donkeys can be significantly enhanced, benefiting both the Animals and the rural communities that depend on them.

## 2. Methodology for Literature Search

This review systematically examines the welfare, management, health, and husbandry practices of donkeys across Asia, focusing on aspects such as feeding, shelter, disease prevention, reproduction, and conservation. To ensure comprehensive coverage and scientific rigor, a structured literature search was conducted across four major databases: PubMed, Web of Science, Scopus, and Google Scholar, covering literature published between 2019 and 2025; a few earlier papers were also included to provide important background and context. The search strategy combined controlled vocabulary and free-text terms using Boolean operators. Core keywords included, donkey, *Equus asinus*, donkey welfare, equine welfare, husbandry, management, nutrition, reproduction, health, disease, vaccination, and preventive medicine, alongside geographical terms such as Asia, South Asia, East Asia, Central Asia, Western Asia, and country names (e.g., Pakistan, India, Afghanistan, China, Iran, Kazakhstan, Uzbekistan, Nepal). Reference lists of key papers and relevant reviews were screened manually to identify additional sources not captured in the primary searches. Only publications in English, or those with an accessible English version, were considered to ensure consistent interpretation.

**Figure 1:**
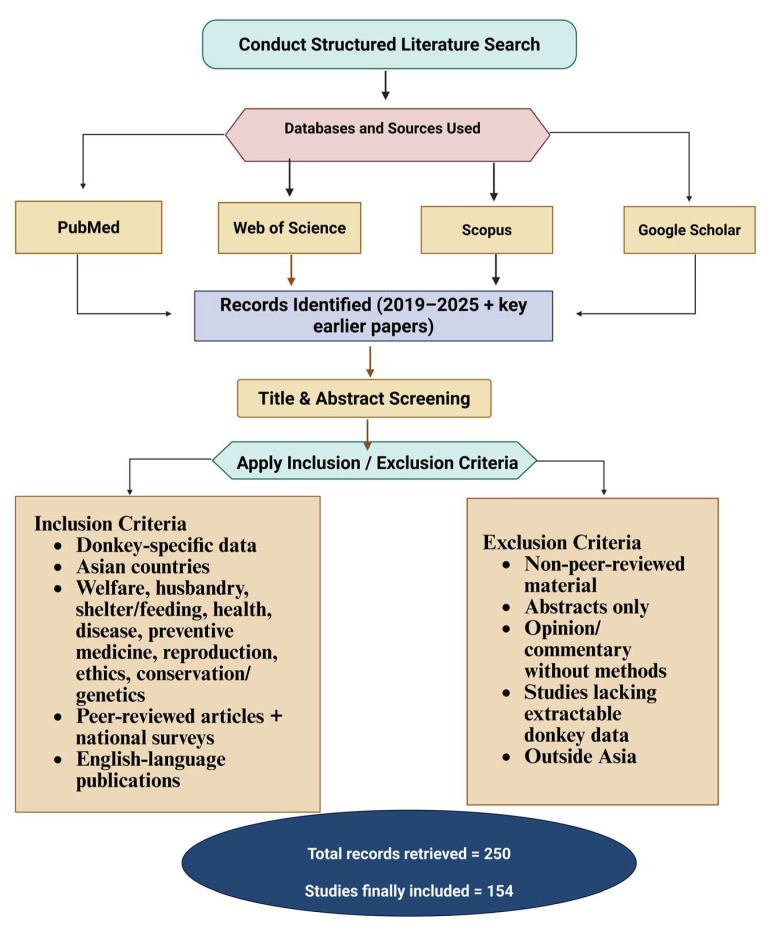
A flowchart of the literature search and article selection.

Records retrieved from the searches were first screened by title and abstract to exclude clearly irrelevant material, followed by full-text assessment against predefined inclusion and exclusion criteria. A total of 250 records were retrieved, and after this screening process, 154 studies were finally included in the review. Studies were included if they reported donkey-specific data from Asian countries and addressed at least one of the following domains: welfare, husbandry and management, feeding and shelter, health and disease, preventive medicine, reproduction and breeding, working conditions and ethics, or conservation and genetic resources. Conference abstracts without full papers, opinion pieces lacking clear methodology, theses not accessible through peer-reviewed channels, and studies focusing solely on horses or wild equids without extractable donkey data were excluded. The final body of literature was then organized by geographic region and thematic focus to enable a coherent, comparative narrative on donkey welfare and husbandry practices across Asia. The literature search methodology and article inclusion and exclusion criteria for this review article are provided in Figure 1.

## 3. Demographics and Population Distribution of Donkeys in Asia

The global population of donkeys is approximately 50 million [2,5], with Africa accounting for the largest share, at 61%. Asia accounts for 26%, while the Americas comprise 13%, and Europe and Oceania collectively represent a mere 0.26% [2,4,5]. The donkey population in South Asia plays a significant role in sustaining rural and semi-urban economies. In Pakistan, the estimated donkey population reached 5.9 million in 2023–2024, with donkeys being essential in provinces such as Balochistan, Khyber Pakhtunkhwa, and Panjab. These Animals are primarily used for transportation, waste management, and agricultural labor, making them integral to local livelihoods in these areas [26,36]. Similarly, India is home to an estimated 1.2 million donkeys, as per the 2019 Livestock Census [37]. In India, donkeys are farmed mainly for load carrying, cart pulling, and transport work, and they remain an important livelihood asset for small and landless farmers. In regions such as Gujarat, donkeys are indispensable for manual labor, particularly in industries like brick kilns, where they transport materials [38]. Additionally, Afghanistan is estimated to have 1.52 million donkeys as of 2023, with Khost Province being a notable area where donkeys play a critical role in livestock farming and rural transportation [39,40]. Across these countries, donkeys serve as vital contributors to Agriculture, transportation, and rural economies.

In Eastern Asia, China has a substantial donkey population, with a recorded 2.68 million donkeys as of 2020, according to the Food and Agriculture Organization [41,42]. By that year, donkeys were primarily concentrated in four provinces: Inner Mongolia (693,000 heads, accounting for 26.65% of the national total), Liaoning (401,000 heads, 15.43%), Xinjiang (361,000 heads, 13.90%), and Gansu (around 208,000 heads, ~8.0% [41,42,43]. Additionally, the total donkey population in China was approximately 2.60 million in 2019, with the majority of donkeys raised in the North (37.7%), Northwest (28.9%), Northeast (18.5%), and Southwest China (11%), while populations in the East (3%) and Central China (0.9%) were minimal [4]. This concentration in specific regions highlights the role of donkeys in supporting agricultural and rural economies in China’s more remote areas.

Historically, traditional donkey breeding farms in China focused on a single breed, but recent surveys identified 16 distinct local donkey breeds across the country. Among them, the Dezhou donkey is the dominant breed, comprising over 57% of China’s total donkey population [41]. This demonstrates the significant role of the Dezhou breed in China’s donkey industry, which is based on both local breeds and crossbreeds raised under a variety of farming systems, including smallholder, semi-intensive, and intensive systems [4]. Unlike many Asian countries, where donkeys are kept mainly as working animals, China has developed a multi-purpose donkey industry centered on the production of ejiao (donkey-hide gelatin), meat, and milk, with large-bodied breeds such as the Dezhou donkey particularly valued for hide quality and carcass traits, while several regional breeds are also used in expanding dairy-focused enterprises [44,45].

China boasts nearly 30 donkey breeds, making it a key player in the genetic diversity of donkeys across Asia [4]. The growing demand for donkey-derived products, such as those used in traditional Chinese medicine (notably ejiao), emphasizes the importance of preserving and managing these genetic resources [46]. The diversity of breeds in China, including Huaibei gray, Liangzhou, Qingyang, Changyuan, Biyang, Gunsha, and others, reflects the nation’s significant contribution to donkey breeding and conservation efforts in Asia [41,42,47].

Other regions in Asia, such as Central Asia, have relatively smaller donkey populations, with Kazakhstan having 29,559 and Uzbekistan 87,974, according to World Population Review (2023) [39]. Despite their smaller populations, donkeys play a crucial role in transport and Agriculture, particularly in remote areas where mechanized transport is limited [48,49]. In the Middle East and Western Asia, including countries like Iran and Iraq, donkeys have been a traditional part of rural life for centuries. Despite the gradual shift towards mechanization, donkeys continue to play a significant role in farming, trade, and transportation, especially in remote or desert regions where motorized vehicles are impractical [50,51,52]. One study reported that donkeys make up nearly 75% of Iran’s total equine population, which is estimated to be around 1.5 million animals, highlighting the country’s significant share of the region’s donkey population [53]. More recent data from the World Population Review (2023) estimates the donkey population in Iran at approximately 1,530,942 (1.53 million) [39]. The estimated global and regional distribution of donkeys, with particular emphasis on Asian countries, is presented in Table 1.

## 4. Donkey Welfare Management in Asia

Proper management and regular care are fundamental to ensuring the health, productivity, and overall well-being of donkeys across Asia. Welfare standards are influenced by multiple interrelated factors, including nutrition, stable and housing conditions, workload intensity, environmental exposure, access to veterinary care, and prevailing cultural attitudes toward animal use [59,60]. In South Asia, particularly in Pakistan and India, working donkeys face significant welfare challenges stemming from poor husbandry and inadequate management practices [61,62]. These conditions often lead to lameness, exhaustion, and reduced productivity. Donkeys employed in sectors like brick kilns endure long working hours and harsh environments, further compromising their welfare [20,63]. Improving donkey welfare in the region requires practical interventions such as training farriers, ensuring balanced nutrition, providing adequate shelter, and promoting community-based healthcare and education programs [61,62]. Strengthening these management practices can enhance both the Animals’ quality of life and the livelihoods of the people who depend on them.

In East Asia, particularly in China, increasing attention to donkey welfare has been driven largely by the rising market demand for donkey-derived products, including ejiao (a traditional Chinese medicine produced from donkey-hide gelatin) [10,64], milk [8,9,65,66,67,68,69], and meat [70,71,72,73,74]. The production of these commodities depends directly on the health, nutrition, and management of donkeys, as optimal welfare is essential for maintaining both product quality and industry sustainability [4,41,42,54]. To meet the growing demand for ejiao, Dong’e Ejiao Co. Ltd. (Liaocheng, China), China’s largest ejiao manufacturer, has established large-scale donkey farms that source hides domestically while promoting welfare-conscious breeding and management practices [45,75]. These regulated farming systems are designed not only to enhance production efficiency but also to improve animal welfare by ensuring appropriate housing, balanced feeding, and veterinary supervision; however, welfare also involves behavioral needs and mental well-being, and the extent to which these systems support social interaction, reduce stress, and meet emotional needs remains unclear.

Additionally, the growth performance of donkeys serves as an important indicator of welfare status. A recent national survey in China reported that donkeys raised on government-managed farms achieved the highest birth weights (over 31.5 kg), reflecting superior nutrition and care standards, whereas those bred on provincial or privately managed farms showed lower birth weights, indicating variations in welfare and management practices across production systems [41]. Although East Asia (China) demonstrates a growing commitment to structured donkey welfare and husbandry management, current welfare standards largely cover intensively farmed donkeys, while rurally owned working donkeys used for transport and agriculture are still managed under traditional conditions without dedicated welfare regulations [45,76]. South Asia continues to exhibit particularly poor welfare management for working donkeys. Furthermore, there is little to no published literature from other Asian nations, such as Central Asia (Uzbekistan, Kazakhstan), Western Asia (Iran, Iraq), and Nepal, indicating a general absence of formal welfare systems in these countries, mainly due to limited research funding and a lack of institutional focus on donkey welfare.

### 4.1. Shelter and Feeding Management of Donkeys in Asia

Appropriate shelter and feeding management are essential aspects of donkey welfare, directly affecting their health, working capacity, and reproductive performance [77,78]. In South Asia, husbandry practices are often inadequate due to limited resources, low awareness, and traditional management systems [14,20]. In Pakistan, for example, most donkey owners do not prioritize proper shelters, particularly in markets and during severe weather, leaving animals exposed to extreme temperatures that cause physical and psychological stress [26]. Wooden or iron-roofed stables are rarely used, and donkeys are typically fed low-quality forages and agricultural by-products that fail to meet their nutritional requirements, resulting in widespread undernutrition. In Balochistan, 34% of donkeys were reported as underweight [26]. Poor grooming, unhygienic conditions, and inconsistent access to water further exacerbate welfare challenges. One study reported that although 64.2% of owners provided food and 58.7% offered water during working hours, these provisions were inconsistent and insufficient [79].

**Figure 2:**
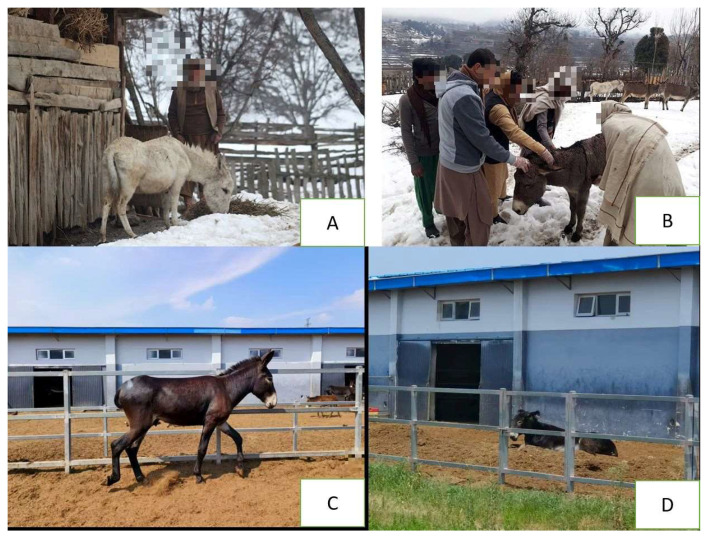
Shelter and feeding management of donkeys in South and East Asia. According to Kamran et al. [26], (**A**,**B**), Traditional donkey management practices in northern Pakistan during winter conditions show limited shelter, water, and feed availability [26]. While (**C**,**D**) Modernized semi-intensive management systems in Dong’e China, where donkeys have access to open paddocks and well-developed shelters, allowing Animals to move freely, rest, and feed comfortably under supervised care. (**C**,**D**) Photograph taken by Changfa Wang.

Comparable constraints are noted in India and Afghanistan. In Gujarat and other Indian states, donkeys are typically tethered outdoors for long durations or housed in makeshift shelters with poor ventilation and sanitation [7,20,38]. Access to clean water and nutritionally balanced feed is limited, particularly in brick kiln industries, where donkeys face long working hours and irregular feeding schedules [20]. In Afghanistan, most donkeys are housed in basic structures built from local materials that provide minimal protection from extreme temperatures, while feeding largely depends on low-quality local forage and crop residues [40]. Collectively, the lack of functional shelters, nutritionally balanced diets, and reliable water access across South Asia highlights the need for owner education and region-appropriate welfare interventions. As illustrated in Figure 2, shelter and feeding management practices for donkeys vary greatly across Asia.

In contrast, China in Eastern Asia has made significant progress toward improving donkey welfare through research-based and semi-intensive management systems, although traditional practices still dominate. Over 70% of Chinese donkeys are raised on smallholder farms under extensive systems, with nearly 45% concentrated in northeastern provinces [80]. A nationwide survey (2021) found that nearly all donkeys (99.5%) were housed throughout the year, but the quality of shelter varied considerably. Most stables were either converted from old residential buildings (44.7%) or constructed as simple brick structures (40.8%), while 93.8% lacked bedding and only 6.2% used straw [54]. A small proportion (0.5%) of donkeys had no shelter at all, raising serious welfare concerns. Experimental research conducted in Inner Mongolia demonstrated that windproof housing significantly improved average daily gain, feed efficiency, and nutrient digestibility during cold weather, underscoring the importance of proper housing for productivity and welfare [81]. Individually owned donkeys in China are managed in simple smallholder systems, kept in basic stables without bedding, fed mainly crop residues, and turned out daily. Routine healthcare is minimal—most receive no vaccination, limited deworming [54]. These findings suggest that although some form of shelter is widely available, upgrading design standards and management consistency remains necessary to enhance welfare outcomes in Chinese donkey farms.

**Figure 3:**
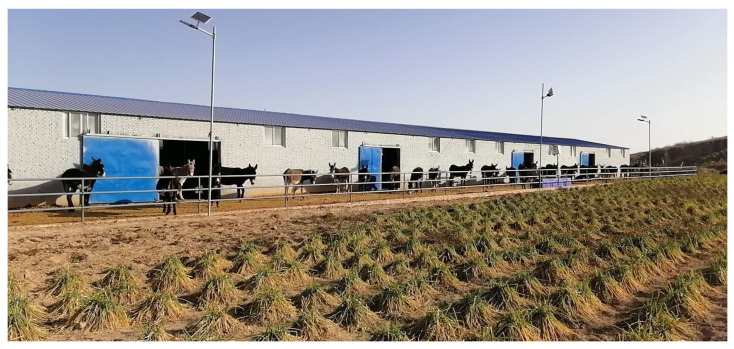
Standard donkey stable constructed in Yulin city, Shaanxi Province, China, illustrating a modern semi-intensive housing system with open paddocks and well-ventilated stables. This facility represents China’s move toward standardized, welfare-oriented donkey husbandry designed to improve comfort, movement, and feeding management. Photograph taken by Changfa Wang.

Feeding management in China reflects a transition from traditional practices toward scientifically informed approaches. Donkeys are primarily fed crop residues such as millet (85.4%) and maize straw (83.1%), supplemented with hay (33.0%) and homemade concentrates including maize (89.2%), soybean meal (16.9%), and sunflower seed meal (14.8%) [54]. Despite these efforts, only 29.0% of farms provide vitamin and mineral supplements, and nearly one-third of donkeys have access only to unclean water sources. Foal management practices, however, show improvement—foals receive colostrum at birth, are fed roughage ad libitum after three months, and are weaned gradually at six to seven months using fenceline separation, reducing stress and morbidity [41]. While traditional feeding methods relying on fibrous roughages like corn silage, peanut vines, and soybean straw remain widespread, an increasing number of farms are adopting total mixed rations (TMR), which have been shown to improve nutrient utilization, gut microbiota balance, and growth performance [82,83]. Overall, donkey shelter and feeding management in China are undergoing modernization, with emerging evidence-based systems promoting improved nutrition, comfort, and welfare. Continued investment in owner training, housing improvement, and nutritional planning will be key to achieving sustainable donkey welfare and productivity across the country. As shown in Figure 3, standard donkey stables built in Shaanxi Province exemplify China’s modern approach to welfare-based housing systems, providing donkeys with improved shelter, ventilation, and access to feed and water.

### 4.2. Health and Disease Management of Donkeys in Asia

**Figure 4:**
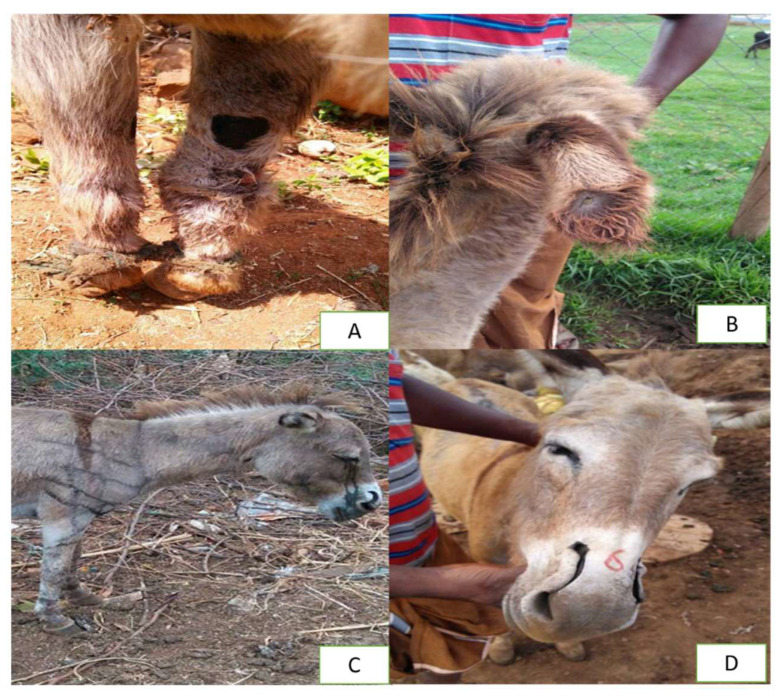
The images illustrate various forms of injuries and mutilations commonly observed in working donkeys across South Asia. Panel (**A**) shows rope-induced wounds on the lower limbs caused by the use of hobbles. Panel (**B**) depicts an ear-splitting mutilation. Panel (**C**) presents multiple branding injuries located on the shoulder and upper limb region. Panel (**D**) displays nasal mutilation involving cuts through both nares, reflecting welfare challenges faced by working equids in the region [86].

Health and disease management remain critical challenges for working donkeys across Asia, particularly in South Asian countries where access to veterinary services is limited and owner awareness is low. In Pakistan, donkeys frequently experience a wide range of health problems, including ocular, dental, and dermatological diseases, as well as lameness and hoof injuries associated with poor farriery practices [26,61,84]. Studies show that 65.3 percent of owners report load-related injuries, with wounds (27.7 percent), lameness (20.5 percent), and back pain (7.2 percent) being the most common conditions [79]. Donkeys working in urban waste collection often display poor body condition, superficial lesions, and signs of muzzle mutilation, underscoring the cumulative effects of physical strain and inadequate care [85]. Donkeys employed in brick kilns also suffer from wounds, overgrown hooves, and distress behaviors due to harsh handling and ill-fitted harnesses [20,38]. Most of these health issues stem from overwork, malnutrition, and the near absence of preventive healthcare such as regular hoof trimming, deworming, and vaccination. Although preventive veterinary measures are essential for managing heat stress, parasitic infestations, and harness-related injuries, their implementation remains inconsistent in both rural and peri-urban settings [7,62]. In Afghanistan, parasitic infections, hoof problems, and malnutrition are common and often left untreated due to limited veterinary outreach [40]. Various forms of injuries and mutilations, including limb wounds, ear and nasal splitting, and branding marks, are also observed in working donkeys across South Asia Figure 4**.**

In Western Asia, similar health challenges persist but are increasingly documented through scientific research. In Iran, several parasitic and infectious diseases have been identified among donkey populations. A study in Ardabil Province reported a 5.5 percent infection rate of Blastocystis sp. The infection was detected in donkeys, with a higher prevalence in diarrheic and younger animals, suggesting possible zoonotic potential and emphasizing the need for improved hygiene and veterinary supervision [87]. Other studies have detected multiple parasitic infections, including Giardia, helminths, and ecto*parasite*s, all of which contribute to digestive and respiratory disorders and indicate the need for enhanced management and deworming programs [88]. Moreover, *Neospora caninum* infection has been detected in 34.5 percent of blood samples and 13.8 percent of aborted fetuses, with evidence of transplacental transmission in 40 percent of aborting jennies, emphasizing the reproductive impact of protozoal diseases and the lack of preventive control measures [89]. Although less common, endocrine disorders have also been reported; for instance, a study on miniature donkeys in Iran described hypothyroidism associated with follicular atrophy, fibrosis, and decreased serum T3 and T4 concentrations, revealing potential gaps in endocrine health research for equids in the region [90]. Despite these documented diseases, preventive care, access to diagnostics, and owner awareness continue to pose major challenges across the region.

In Eastern Asia, recent investigations have revealed that donkey populations in China face a complex array of health challenges associated with infectious, parasitic, and metabolic diseases, as well as insufficient preventive care and owner awareness [42,54]. Numerous studies have documented reproductive disorders and widespread viral, bacterial, and parasitic infections affecting donkeys across various regions [91,92,93,94]. China’s expanding donkey industry has prompted strategic investments in disease surveillance, which have uncovered diverse pathogens posing serious risks to both Animal Welfare and public health [13]. Among the viral diseases, Equid herpesvirus 8 (EHV-8) has received particular attention for its pathogenic potential. Experimental models using C57BL/6J mice demonstrated that EHV-8 infection causes clinical symptoms such as dyspnea, weight loss, and viremia, accompanied by elevated pro-inflammatory cytokines (IL-6, IL-1β, TNF-α) in brain and lung tissues [95,96]. Subsequent studies revealed that the antioxidant enzyme heme oxygenase-1 (HO-1) can suppress EHV-8 replication via PKCβ/ERK1/2 and NO/cGMP/PKG signaling pathways, with biliverdin identified as a key inhibitory metabolite [97]. Promising therapeutic advances have been reported using bioactive compounds such as rutin, blebbistatin, hyperoside, and cobalt protoporphyrin (CoPP) in controlling viral replication [98,99,100,101]. Beyond herpesviruses, donkeys in China have been found to harbor Hepatitis E virus (HEV) genotypes 3 and 4 and a novel astrovirus (DAstV-1) linked to severe neonatal diarrhea, both of which carry notable zoonotic implications for human health [102,103].

Parasitic and bacterial infections also represent major health threats to Chinese donkey populations. Studies have reported high infection rates of *Giardia duodenalis*, *Theileria equi*, *Babesia caballi*, *Enterocytozoon bieneusi*, and several *Entamoeba* species across multiple provinces [104,105,106,107]. In Inner Mongolia, three *Cryptosporidium s*pecies were identified alongside *Giardia* and *Enterocytozoon,* emphasizing their co-occurrence and potential for cross-species transmission [108]. These *Parasite*s, prevalent in Xinjiang, Gansu, and Inner Mongolia, are recognized causes of gastrointestinal illness in both donkeys and humans, posing significant public-health concerns [93,108]. *Enterocytozoon bieneusi* has been reported from Shandong, Jilin, and Liaoning provinces [106], while *Toxoplasma gondii* has been isolated from donkey serum, meat, and milk, highlighting risks to pregnant women and immunocompromised individuals [109,110]. Despite the expanding knowledge base, routine veterinary interventions remain limited in many areas. A nationwide survey in northeastern China revealed that 37.9 percent of donkeys were not dewormed annually, 44.5 percent received deworming once per year, and only 12.4 percent twice per year. None of the Animals in the study had received vaccinations or dental care, and 14.8 percent had never undergone hoof trimming [54]. The same survey reported colic (13.5 percent), respiratory disorders (10.9 percent), skin conditions (9.2 percent), lameness (3.4 percent), and dental disorders (2.7 percent) as the most frequent medical issues [54]. As shown in Table 2, welfare challenges differ across Asia, with inadequate shelter, nutrition, and veterinary care most pronounced in South Asia, and more structured management systems present in China.

Emerging genomic and molecular studies have advanced the understanding of infectious disease mechanisms in Chinese donkeys. Whole-genome sequencing recently characterized equine coronavirus isolates from diarrheic donkeys in Shandong Province [111]. Additional research on reproductive health revealed that Streptococcus zooepidemicus strains from jennies with infertility carried more virulence genes than those from healthy animals [112]. Proteomic analyses have also identified differentially expressed serum proteins in donkeys with Escherichia coli-induced endometritis, suggesting novel biomarkers for diagnosis and disease monitoring [113]. Although these advances are promising, omics-based approaches in donkey medicine remain in their infancy, warranting further exploration of genetic and proteomic tools for disease prevention and early detection [12]. Although these developments represent significant scientific progress, routine preventive care and veterinary monitoring remain limited, indicating the need for stronger surveillance, expanded owner education, and integrated health-management strategies.

**Table 2:** Major welfare challenges and management practices affecting donkeys across Asia.

Region/Country	Welfare Improvement Challenges	Preventive Healthcare Practices	Welfare Improvement Measures	References
Pakistan	Poor shelter, inadequate nutrition, lack of veterinary care, overloading, wounds, hoof problems	Low vaccination and deworming rates; minimal preventive care awareness	Owner education, shelter improvements, farriery training, veterinary access	[26,79]
India	Overworking in brick kilns, poor shelter, irregular feeding, lack of veterinary services	Limited vaccination (tetanus, influenza); poor access to vets	Improved feeding, water access, shelter, community training programs	[7,20,38]
Afghanistan	Basic shelters, poor feed quality, parasitic infections, limited vet outreach	Minimal vaccination; absence of national programs	Awareness campaigns, improved feed and shelter, training for farmers	[40]
China	Housing deficiencies, limited vaccination, parasitic and viral infections, inconsistent feeding standards	Low vaccination (none in surveyed farms); 37.9% no deworming	Structured welfare programs, improved housing, adoption of total mixed ration feeding, biosecurity reinforcement	[54,80,114,115]
Iran	Parasitic infections (Giardia, helminths), reproductive losses (Neospora caninum), and limited welfare research	General livestock vaccination applies to equids; limited equine-specific programs	Enhanced hygiene, targeted *Parasite* control, better reproductive monitoring	[87,88]
Central Asia (Uzbekistan, Kazakhstan)	Small populations, limited management systems, and minimal welfare documentation	Not well documented; presumed minimal preventive care	Regional welfare documentation and management program development	[116,117]

### 4.3. Vaccination Strategies

The management and medical treatment of donkey diseases across Asia remain largely inadequate, with preventive health measures such as vaccination, deworming, and routine veterinary care being severely underutilized [7,26]. A participatory appraisal conducted in Balochistan, Pakistan, revealed that most donkey owners had minimal awareness of preventive interventions and lacked access to affordable or well-coordinated veterinary services [26]. Although vaccination is recognized as a cornerstone of animal health management, few owners understand which vaccines are appropriate or available, and preventive practices are rarely implemented. These deficiencies extend beyond infectious disease control, as addressing work-related injuries should also be integrated into broader preventive health strategies [79]. In India, vaccination programs targeting common equine diseases such as tetanus, equine influenza, and strangles are critical for improving donkey welfare; however, implementation remains limited due to poor access to veterinary infrastructure and low awareness among owners [7,62]. Consequently, many working donkeys remain unvaccinated and susceptible to preventable diseases. Similarly, in Afghanistan, vaccination rates are extremely low, primarily due to limited veterinary services and the absence of organized national programs promoting equine vaccination. Preventive medicine against tetanus, rabies, and equine influenza is especially important for reducing mortality and disease transmission in these populations [40]. In Western Asia, particularly Iran, specific studies on donkey vaccination are scarce; however, general livestock vaccination protocols practiced in the country are presumed to extend to equids, suggesting a foundation upon which species-specific preventive programs could be developed [118].

In Eastern Asia, a national survey conducted in China by Deng et al. [54] revealed serious deficiencies in routine healthcare: 37.9% of donkeys were not dewormed annually, 44.5% received deworming once per year, and only 12.4% twice per year; notably, none of the animals examined had ever been vaccinated or received dental care from a veterinarian or technician. This lack of vaccination and basic preventive care increases the risk of infectious disease outbreaks and negatively affects both welfare and productivity. Economic barriers further exacerbate these gaps, as the high costs of essential equine vaccines, such as those for equine influenza and tetanus, make regular immunization financially unfeasible for many smallholder and private farms [114,115]. Wang et al. [115], reported that while vaccination and preventive measures are critical for improving foal survival and herd health, most Chinese farms have yet to establish routine vaccination programs due to their expense and limited veterinary infrastructure. Without systematic immunization, donkeys remain highly susceptible to vaccine-preventable diseases, threatening both animal health and the economic sustainability of donkey-based industries.

Recent research highlights the growing importance of integrating vaccination into comprehensive preventive medicine frameworks in China. A 2023 study documented widespread infections of Equine herpesvirus (EHV-1 and EHV-4) in large-scale donkey farms in Liaocheng, demonstrating their significant impact on immune function and oxidative balance [119]. The study emphasized the urgent need for prophylactic immunization, strengthened biosecurity, and seasonal management strategies to reduce viral transmission, while calling for the development of donkey-specific EHV vaccines. Similarly, molecular and epidemiological studies have identified numerous pathogens such as *Giardia duodenalis*, Cryptosporidium spp., *Enterocytozoon bieneusi,* and *Toxoplasma gondii* across several provinces, reinforcing the importance of preventive health planning and disease control [102,106]. Despite these findings, routine vaccination rates remain extremely low, indicating a critical gap between research advances and field implementation. A 2025 study further emphasized the importance of preventive neonatal care, recommending tetanus vaccination for jennies, antitoxin administration for newborn foals, and strict hygiene and colostrum-monitoring protocols to reduce early-life morbidity and mortality [120]. Collectively, these studies underline that effective preventive medicine incorporating affordable vaccination, regular deworming, biosecurity, and neonatal health protocols is vital to improving welfare standards, disease resistance, and sustainable productivity in China’s modern donkey industry.

## 5. Reproduction and Breeding Practices

Effective reproductive management plays a pivotal role in donkey production systems, as it determines herd expansion, ensures genetic conservation, and underpins the long-term economic sustainability of the industry. Recent studies have highlighted that advancements in assisted reproductive technologies (ARTs), such as artificial insemination (AI) and embryo transfer (ET), are essential tools for enhancing fertility, preserving genetic resources, and improving productivity in donkey breeding programs [121]. Donkeys (*Equus asinus*) are generally nonseasonal, polyestrous breeders with an estrous cycle averaging 21–28 days and an estrus duration of 6–9 days [122,123]. In Pakistan, breeding is primarily by natural service, with limited application of AI due to financial, technical, and infrastructural constraints. However, hormonal induction using GnRH, hCG, and prostaglandin (PG) has been shown to effectively synchronize estrus and ovulation, achieving pregnancy rates of up to 80 percent in American Mammoth Jackstock jennies under subtropical conditions [124]. Although such protocols are not yet widely applied in Pakistan, they demonstrate the potential of reproductive biotechnologies to improve conception rates under field conditions. Responsible breeding management should also emphasize long-term ownership and care, fostering stronger human–animal relationships that enhance donkey welfare and behavioral stability. Moreover, implementing structured breeding programs is vital to prevent inbreeding and maintain genetically healthy populations [62]. These reproductive interventions also have welfare implications, as hormonal synchronization, semen collection, and intensive breeding schedules may increase handling stress and limit natural behaviors, whereas controlled mating systems can reduce injuries commonly seen in unregulated natural service.

Across South and Central Asia, reproductive practices in donkeys remain largely traditional and poorly organized, resulting in low productivity and limited genetic progress [20]. In India, breeding programs are typically informal, with donkeys maintained mainly for labor rather than for selective genetic improvement. Consequently, breeding decisions are driven by utility rather than reproductive performance, although recent efforts have aimed to introduce sustainable breeding and management practices to improve the health and longevity of working donkeys [7]. In Afghanistan, donkey reproduction is almost entirely based on traditional methods, characterized by unregulated mating within local herds and minimal record keeping. The absence of selection for desirable traits, combined with limited veterinary input, leads to reduced fertility, low foal survival rates, and a narrow genetic base [40].

To ensure the long-term sustainability of donkey populations, responsible breeding practices are essential. Promoting long-term ownership and proper management helps strengthen human–animal bonds, which in turn improves welfare, behavior, and overall reproductive performance. Responsible breeding is also important for preventing inbreeding and maintaining healthy genetic diversity within local populations [62]. In India, breeding practices remain largely traditional, with donkeys being maintained primarily for labor rather than for genetic improvement or reproductive performance.

However, some regions are beginning to incorporate more sustainable breeding practices to improve the health and longevity of working donkeys [7]. Collectively, these challenges underscore the urgent need for structured breeding policies, reproductive monitoring, and veterinary extension programs across the region. Promoting scientifically informed breeding practices, supported by hormonal management and reproductive technologies, can substantially enhance reproductive efficiency, safeguard genetic diversity, and ensure the long-term sustainability of donkey populations in Asia.

**Figure 5:**
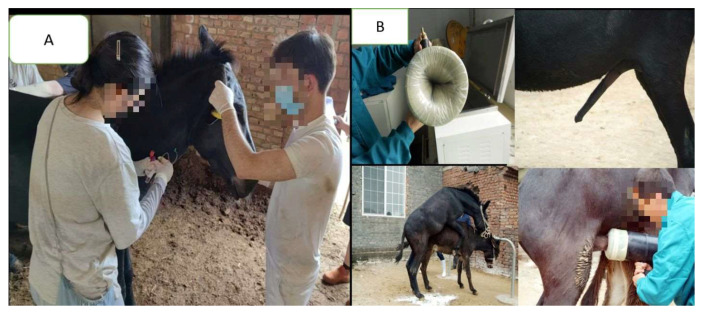
Reproductive management and breeding evaluation of Dezhou donkeys in China, showing (**A**) blood sample collection for genetic and reproductive assessment and (**B**) Semen collection and processing procedures, including preparation of the artificial vagina, stimulation of the male donkey, and collection of ejaculate for evaluation and artificial insemination. These steps represent standardized reproductive management practices aimed at improving fertility, genetic selection, and sustainable breeding efficiency in the Dezhou donkey population. Photograph taken by Changfa Wang.

In Eastern Asia, particularly in China, donkey reproduction and breeding have evolved rapidly in response to rising demand for donkey-derived products such as ejiao, milk, and meat. To stimulate the industry, 15 provinces and 22 cities have recently announced financial subsidies to promote large-scale donkey breeding initiatives [125]. Chinese donkey farming primarily relies on indigenous breeds, with the Dezhou donkey being the most preferred due to its superior body size, reproductive performance, and high-quality hides suitable for ejiao production [94,126]. Selective breeding within these programs ensures genetic improvement and the sustainability of local populations. Advancements in artificial insemination (AI) have greatly improved fertility outcomes [127]. At the National Black Donkey Breeding Center, Dezhou donkeys achieved remarkable progress, with conception rates rising from 45% in 2013 to 92% in 2016, following improved AI techniques and semen management [45,127]. Intensive breeding systems are now well established, relying on structured female breeding stocks and scientifically managed reproduction [75,128]. A large-scale survey of 38 breeding farms reported that 73% used AI, with better reproductive performance observed in provincial and national farms compared to self-owned operations. Average age at first semen collection was 3.1 years, jennies foaled at around 39 months, and foaling intervals averaged 410 days, highlighting the impact of professional management and breed selection on reproductive success [41]. As shown in Figure 5, modern reproductive management in China involves both genetic evaluation through blood sampling and semen collection for artificial insemination at Dong’e, reflecting the country’s growing emphasis on scientifically guided breeding programs to enhance reproductive efficiency and genetic improvement in Dezhou donkeys.

Donkey breeding farms in China are categorized as national, provincial, and local farms, mostly concentrated in the northern regions, where 52% of farms maintain 100–500 donkeys, while only a few exceed 2000 animals [41,54]. Educational level and technical expertise of farm managers strongly influence breeding efficiency, as well-trained managers adopt better welfare, nutrition, and veterinary practices [41,54]. Both natural mating and AI are widely used, though AI dominates in large-scale farms and contributes to conception rates as high as 70–92% in Dezhou donkeys [41,129,130]. Recent studies have also explored inhibin immunization to improve reproductive efficiency and hormonal balance during non-breeding seasons [131]. The ongoing transition from traditional smallholder systems to large-scale, integrated industrial farms reflects China’s commitment to modernizing donkey reproduction while ensuring Animal Welfare and sustainability. However, the adoption of advanced technologies, particularly AI with frozen semen and embryo transfer, remains uneven across regions, and wider application of these methods could further improve reproductive efficiency, herd genetics, and the long-term stability of China’s donkey population [115,132]. China has made substantial progress in donkey reproduction through assisted reproductive technologies, structured breeding programs, and improved management, leading to notable gains in fertility and productivity. In contrast, much of South and Central Asia still rely heavily on informal natural service and unmanaged herd mating, which restricts reproductive efficiency and slow genetic improvement. Continued expansion of modern reproductive practices across diverse production systems is essential to support long-term herd development and welfare.

## 6. Donkey Overwork and Ethical Considerations in Asia

In South Asia, particularly in Pakistan, India, and Afghanistan, donkey welfare is deeply intertwined with ethical considerations that reflect both socioeconomic realities and cultural perceptions. In Pakistan, working donkeys face severe welfare challenges caused by overloading, poor farriery practices, and inadequate access to basic healthcare, all of which result in chronic pain, injury, and reduced working lifespan [61,84]. Addressing these welfare issues is an ethical necessity, requiring improved farriery training, increased owner education, and accessible veterinary services to prevent avoidable suffering. Encouragingly, research shows that awareness of donkey sentience directly influences welfare practices: 81.3% of Pakistani owners acknowledged that donkeys feel pain, and 70% recognized their emotional capacity, suggesting that empathy-based education can play a vital role in improving management and care standards [79]. Promoting such understanding is essential to building ethical responsibility among owners and ensuring that working donkeys are treated with respect and compassion rather than merely as labor assets.

**Figure 6:**
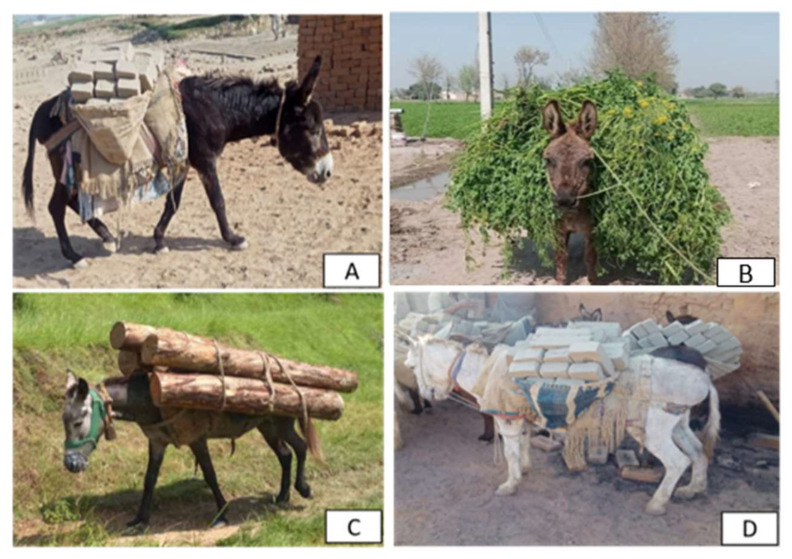
The overloading and working conditions of donkeys. (**A**) A donkey carrying heavy brick loads in a brick kiln industry [14], (**B**) A donkey transporting bulky green fodder loads from fields to households [135], (**C**) a donkey transporting logs in rural terrain [19], and (**D**) multiple donkeys carrying excessive brick loads in kiln environments [136]. All examples demonstrate the physical strain and welfare challenges caused by excessive workloads commonly observed among working donkeys across South Asia.

Ethical concerns extend across South Asia, where donkeys are often perceived primarily as tools for labor rather than sentient beings. In India, studies from Gujarat and the brick kiln industry highlight that donkeys frequently endure harsh working conditions, including physical punishment, overexertion, and neglect of basic needs [20,38]. These practices raise serious ethical questions about the moral obligations of owners toward their animals and emphasize the urgent need for welfare education and enforcement of humane standards. Social inequities also compound these challenges: donkey ownership in some regions is linked to marginalized caste groups, who face economic hardships that limit their ability to provide adequate care [7,62]. In Afghanistan, ethical considerations are equally critical, as donkeys form the backbone of rural transport systems, yet remain highly vulnerable due to poverty and the absence of veterinary infrastructure [40]. As shown in Figure 6, working donkeys in South Asia often experience severe overloading and prolonged physical exertion remains common across South Asia, resulting in fatigue and injury [14,85]. Across South Asia, improving welfare standards for working donkeys requires not only technical interventions but also a moral and cultural shift—recognizing donkeys as sentient, emotionally aware beings deserving of humane treatment and protection under ethical and welfare frameworks.

In China, ethical considerations surrounding donkey welfare are increasingly recognized within the context of modernization, commercial use, and intensive breeding. Feeding management plays a crucial role in maintaining both physiological health and ethical husbandry standards. Liu et al. (2020) reported that reducing the forage-to-concentrate (F/C) ratio below 55% adversely affects nutrient utilization, nitrogen balance, and metabolic health in donkeys, leading to poor body condition, reduced fertility, and increased susceptibility to metabolic disorders such as laminitis and hyperlipemia [133]. Ethical debates in China also center on the commercial exploitation of donkeys, particularly in relation to the ejiao (donkey-hide gelatin) industry. Research conducted in 2020 and 2023 highlights widespread public concern regarding the inhumane treatment of donkeys during transport and slaughter for hide extraction, driven by increasing domestic and international demand for ejiao [45,134]. This has led to ethical dilemmas regarding the treatment of donkeys as economic commodities rather than sentient *Animals*. Although Deng et al. (2021) [54], reported that most donkeys in northeastern China are bred for reproductive or labor purposes (79.7% for breeding, 69.1% for draft work, and only 11.4% for meat or hide production). Ensuring ethical donkey welfare in China depends on balancing industry growth with humane treatment and recognizing donkeys as sentient *Animals* deserving proper care and respect.

Ethical risk drivers differ by region. In South Asia, overloading, harsh handling, and injury are primarily linked to poverty-based dependence on donkeys for daily labor, meaning that welfare improvement depends on owner education, farriery training, and accessible community veterinary services [14,20,38,61,79,84,85]. In Eastern Asia, China, ethical concerns increasingly arise from commercialization and the ejiao trade, requiring stronger regulation of transport, feeding intensity, and slaughter standards [45,54,133,134]. Central and Western Asia remain poorly studied in ethical terms, suggesting that welfare awareness and policy frameworks are not yet fully developed or documented.

## 7. Donkey Conservation Efforts in Asia

Conservation efforts for donkeys in South Asia are closely linked to improving their welfare, as maintaining healthy working populations is essential for ensuring long-term sustainability. In Pakistan, although formal conservation initiatives are limited, improving donkey welfare through better farriery, healthcare, and owner education has been recognized as a key component of population preservation [84]. Community-based interventions that integrate veterinary support, poverty alleviation, and social empowerment for owners are crucial for sustaining both donkey populations and the livelihoods that depend on them [62]. In India, efforts to conserve donkeys remain fragmented but include initiatives to enhance health management, raise public awareness about the species’ importance, and promote ethical breeding practices to ensure genetic diversity and long-term viability [7]. In Afghanistan, formal conservation programs are scarce, yet increasing recognition of the donkey’s role in rural economies has encouraged interest in preserving and managing local populations more responsibly [40]. Overall, while South Asian countries have made some progress through welfare-centered approaches, structured donkey conservation programs remain underdeveloped, and other Asian regions currently lack comprehensive information or documented efforts focused on donkey conservation.

Globally, the donkey population is declining not only due to inadequate conservation initiatives and reduced utilization of donkey-derived products but also because of increased mechanization, limited policy attention [4,12,137,138]. In contrast, in Eastern Asia, China has emerged as a regional leader in donkey research, with growing emphasis on sustainable breeding, welfare, and genetic conservation; however, the formal concept of ‘donkey welfare’ entered China relatively late, around 2019 onward [4,12,41,42,54,137]. Mechanization has reduced traditional uses of donkeys in traction and transport [45]. The Chinese government and research institutions have placed increasing emphasis on improving management systems, breed development, and production efficiency [4,41,54,104]. National surveys indicate the presence of well-equipped breeding farms, with China currently conserving 24 indigenous breeds, ranking first worldwide in donkey genetic resource preservation [4]. Donkeys are now reared primarily for milk, meat, and hide, driving both scientific and commercial interest [41,66,67,139]. To sustain this progress, the government has introduced subsidy programs and policy incentives to expand breeding and strengthen the industry [45]. Large-scale breeding farms—mainly in northern, northwestern, and northeastern China—now represent over 13% of the national population, serving as key centers for production and genetic conservation [140].

**Figure 7:**
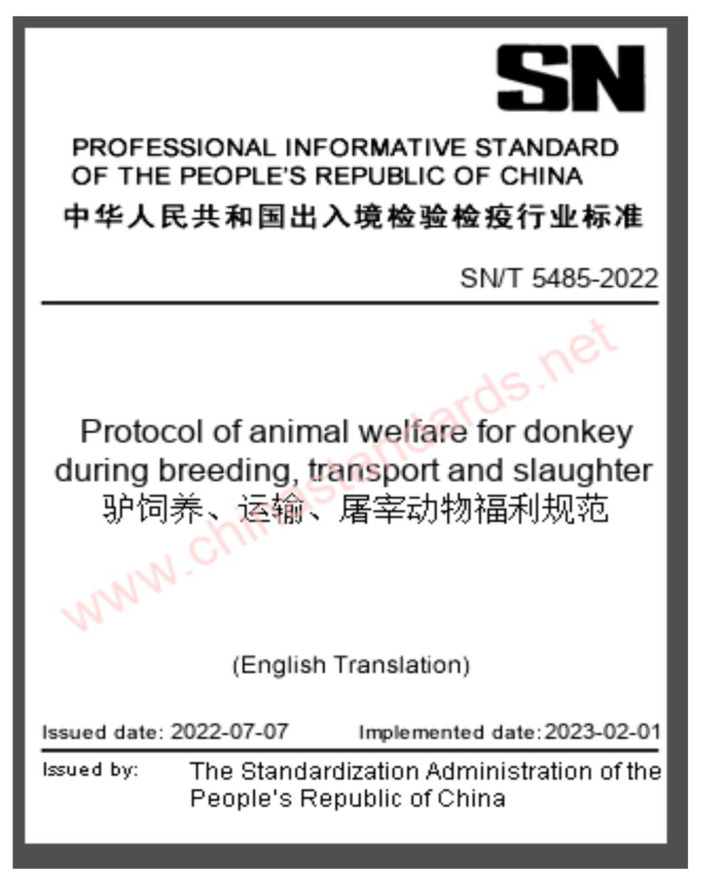
Cover page of the Chinese national industry standard Protocol of Animal welfare for donkeys during breeding, transport and slaughter (SN/T 5485-2022), issued by the Standardization Administration of the People’s Republic of China in 2022. The document establishes formal welfare requirements for donkey breeding, transport, and slaughter management, reflecting China’s increasing institutional emphasis on equine welfare [142].

The Ministry of Agriculture and Rural Affairs has prioritized the protection of animal genetic resources to prevent the erosion of valuable donkey germplasm and ensure long-term sustainability [141]. Both government agencies and private enterprises have developed a multi-tier conservation network, including national and provincial breeding centers dedicated to maintaining genetic diversity [41]. The issuance of the national welfare standard SN/T 5485-2022 (Figure 7) represents a milestone in China’s formal recognition of donkey welfare, integrating housing, handling, transport, and slaughter procedures under standardized regulations [142].

**Figure 8:**
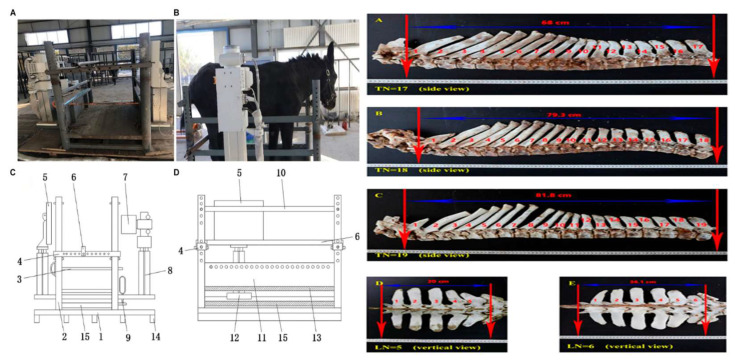
Radiography bed design and vertebral variation in Dezhou donkeys. (**A**–**D**) The **left** panels show the physical and schematic models of the radiography bed, including front and side views that highlight the main structural components used for accurate positioning and radiographic imaging of donkeys. (**A**) Front view of the physical bed; (**B**) side view of the physical bed; (**C**) model front view; (**D**) model side view. (**A**–**E**) The **right** panels illustrate morphological variation in thoracic and lumbar vertebrae of Dezhou donkeys, demonstrating differences in vertebral number and structure that indicate genetic diversity within the breed. (Adapted from Liu et al. [144], Wang et al. [153], and Khan et al. [148]).

Recent advances in molecular genetics have provided new tools for the conservation and sustainable improvement of donkey breeds. Studies by Liu Z. et al. [143] involving 455 Dezhou donkeys revealed significant variability in thoracic and lumbar vertebral configurations (T18L5, T18L6, T17L6, T17L5, and T19L5), with T18L5 being the most common (75.8%), reflecting genetic diversity within the population. These structural differences were correlated with body size and weight [143,144]. Further research identified key genes, such as *PRKG2*, *NR6A1*, *LTBP2*, *HOXC8*, and *DCAF7*, as crucial determinants of vertebral number and length [11,137,143,144,145,146,147,148]. Additional loci, including *NLGN1*, *DCC*, *FBXO4*, *SLC26A7*, *TOX*, *LRP5*, *WNT7A*, LOC123286078, LOC123280142, GABBR2, LOC123277146, LOC123277359, BMP7, B3GAT1, and EML2, are involved in Wnt and TGF-β signaling pathways regulating embryonic and bone development [149]. Collectively, these findings provide a genomic foundation for marker-assisted selection and the preservation of structural and adaptive traits in donkeys. Figure 8. Radiography bed design and vertebral variation in Dezhou donkeys illustrate the morphological diversity underpinning these conservation developments.

Importantly, these genetic findings have direct practical relevance for breeding and conservation efforts [150]. Genes associated with vertebral traits and body size offer measurable markers that can be incorporated into marker-assisted selection to improve growth, skeletal soundness, conformation, and production performance in breeding programs [148]. For conservation centers, genomic data help identify genetically distinct lineages, monitor inbreeding, and guide mating decisions to maintain genetic diversity. Thus, integrating molecular markers into routine herd management provides breeders with actionable tools for selecting superior animals, while helping conservation planners safeguard rare genotypes and prevent genetic erosion.

Advances in molecular genetics are further supporting these goals. A 2024 study employed a 40K liquid SNP chip to analyze genetic variation in Dezhou donkeys, representing a significant step toward genomic-level conservation [151]. Meanwhile, the rapid growth of donkey milk farms reflects the integration of economic development with conservation objectives. However, the donkey dairy sector remains at an early stage and exhibits regional differences between eastern and western areas [43,152]. It has generated considerable benefits in pastoral regions such as Xinjiang, where dairy production helps sustain local livelihoods and promotes population stability [43]. Overall, China’s integrated approach combining policy support, genetic preservation, and sustainable industry development demonstrates a strong national commitment to conserving and improving donkey populations in Eastern Asia.

Conservation efforts remain uneven across Asia. In South Asia, conservation is largely indirect and welfare-centered, relying on improving health, farriery, and owner awareness rather than formal population-level strategies [7,40,62,84]. China has adopted a more integrated pathway combining policy incentives, breed-resource infrastructure, welfare standards, and genomic selection to preserve indigenous breeds and expand sustainable industries [4,12,41,42,54,137,149,151]. By contrast, Central and Western Asia lack comprehensive conservation documentation, indicating a critical regional priority for baseline surveys, breed registries, and coordinated conservation policy.

## 8. Recommendations for Improving Donkey Welfare in Asia

Improving donkey welfare in South Asia, particularly in Pakistan, India, and Afghanistan, requires a multifaceted approach that combines education, veterinary care, farriery training, and sustainable management. In Pakistan, targeted educational programs are needed to reduce overloading and promote humane handling practices, ensuring that working donkeys are not subjected to excessive physical strain [14]. Tailored management strategies should be integrated into livestock development programs, supported by veterinarians and welfare organizations distributing guidelines in local languages to raise awareness among owners [26]. Structured vaccination programs, improved farriery, and preventive healthcare are essential for controlling diseases and extending working life [61,84]. Studies also recommend that donkey owners provide adequate food and water during work hours, use properly padded saddles, and learn to recognize signs of pain or distress to prevent unnecessary suffering [79]. In India, welfare improvement must focus on shelter and environmental protection, ensuring consistent access to clean water and feed, and implementing regular veterinary checks, including hoof care and vaccinations, to prevent injury and disease [38]. Moreover, community-based programs that empower donkey owners through training and financial support can strengthen welfare outcomes and sustain healthy donkey populations [62].

Across Asia, improving donkey welfare also depends on broader socioeconomic and policy interventions. In Afghanistan, raising awareness among farmers through educational campaigns on feeding, shelter, and veterinary care is critical to address the neglect that often stems from poverty and lack of knowledge [40]. In China and other regions, efforts should prioritize better housing conditions, access to water and feed, routine healthcare, and the regulation of working conditions to reduce overexertion and abuse [20]. Promoting sustainable alternatives to donkey labor, such as mechanization in heavy industries, can further minimize exploitation while preserving donkey welfare [20,62]. A “One Welfare” approach—linking human, animal, and environmental well-being—should guide welfare improvement policies, particularly in developing areas where human livelihoods are closely tied to donkey labor [62]. Strengthening collaboration among governments, NGOs, and local communities, combined with policy enforcement, owner education, and access to affordable veterinary services, will ensure a more ethical, sustainable, and welfare-oriented future for donkeys across Asia [7,38].

In China, the future of the donkey industry depends on a coordinated approach that integrates genetic conservation, welfare improvement, and sustainable commercialization. Establishing regional characteristic brands can enhance consumer awareness and trust, while applying modern marketing strategies, including improved distribution networks and brand promotion, can expand market share and strengthen industry competitiveness [43,154]. Governmental policy support is equally vital to promote sustainable development, encourage innovation, and facilitate collaboration across the entire industrial chain, linking breeding, production, and product processing to reduce costs and improve efficiency [43,154]. To ensure long-term progress, urgent attention must be given to preserving the genetic diversity of indigenous breeds, improving large-scale breeding and production management, and enhancing healthcare, nutrition, and product quality to maintain both profitability and animal welfare [41,54]. Implementing quality control and traceability systems for donkey-derived products, alongside farmer education and sustainable husbandry practices, will further ensure ethical treatment, stable income generation, and the long-term sustainability of China’s growing donkey industry [4]. A summary Table 3 has been included to consolidate the core information on reproduction and breeding practices, overwork and ethical concerns, conservation status, and welfare recommendations across Asian regions.

**Table 3:** Summary of reproductive practices, key welfare concerns, and conservation drivers affecting donkeys across major Asian regions.

Region/Country	Reproduction and Breeding Practices	Overwork and Ethical Concerns	Conservation Status and Key Drivers	References
Pakistan	Natural service is predominant; AI is rarely used due to financial and technical constraints. Hormonal induction (GnRH, hCG, PG) is effective but not applied widely. Low genetic selection and unstructured breeding programs.	Severe overloading, poor farriery, and inadequate access to veterinary care. Welfare affected by poverty; owners show partial awareness of donkey pain and emotions	No formal conservation programs; population threatened by neglect, disease, and mechanization. Conservation is mainly achieved indirectly through welfare improvement.	[7,14,61,79,84,85]
India	Traditional, utility-driven breeding. Little use of selective breeding or reproductive technologies. Weak record-keeping.	Brick-kiln donkeys exposed to harsh conditions, punishment, poor nutrition, and extreme workloads. Ethical concerns tied to marginalized socioeconomic groups.	Fragmented conservation efforts; limited documentation. Mechanization and social marginalization contribute to population decline.	[20,38,62]
Afghanistan	Entirely traditional and unregulated mating. No structured breeding programs; minimal veterinary involvement; low foal survival rates.	Heavy dependence on donkeys due to poverty; widespread neglect, overloading, and lack of healthcare.	Very limited conservation documentation; population vulnerable due to poverty and conflict.	[40]
China	Highly modernized breeding sector. AI widely used in large farms (70–92% conception rates). Genomic tools, hormonal protocols, and structured breeding centers established. Strong government subsidy support.	Ethical issues linked to the ejiao industry and transport/slaughter conditions. Nutritional mismanagement may lead to metabolic disorders (laminitis, hyperlipemia).	Strong national conservation framework: 24 indigenous breeds preserved; multi-tier breeding centers; formal welfare standard (SN/T 5485-2022) implemented. Mechanization reduces traditional use.	[41,43,54,125,141,142]

## 9. Conclusions

Donkeys remain indispensable to Agriculture, transport, and livelihoods across Asia; however, welfare investment, preventive healthcare, and ethical governance continue to fall short of what is needed. In South Asia, particularly in countries such as Pakistan, India, and Afghanistan, donkeys face serious welfare challenges, including poor nutrition, lack of shelter, inadequate farriery, and limited access to veterinary services. These conditions often result in overwork, high disease incidence, and low productivity. Central and Western Asia remain poorly documented, but available evidence indicates similar welfare constraints driven by limited veterinary infrastructure and the near absence of formal welfare policy. In contrast, China presents a more structured and organized approach that integrates policy support, welfare-oriented husbandry, and research-driven breeding programs. Recent advances in genomic studies, especially those identifying genes related to vertebral number, skeletal development, and body conformation in Dezhou donkeys, have created new opportunities for conservation and selective breeding, allowing welfare, productivity, and conservation goals to be aligned more effectively.

A sustainable way forward is to adopt a “One Welfare” approach that integrates animal welfare, human livelihoods, and environmental health through affordable healthcare, better housing and nutrition, and improved owner awareness. Furthermore, enforcing welfare standards across working, transport, and slaughter systems, along with developing regional genetic databases and expanding reproductive technologies, will strengthen both welfare and productivity. Ultimately, combining practical husbandry with accessible veterinary care, ethical oversight, and genomic innovation provides a comprehensive and scalable framework for ensuring the long-term conservation, health, and economic value of donkeys across Asia. A notable limitation identified in this review is the scarcity of published research addressing the mental and affective dimensions of donkey welfare, as most existing evidence remains focused on physical health and management indicators; this gap underscores the need for future studies exploring cognitive–emotional well-being in donkeys.

## Figures and Tables

**Table 1:** Estimated donkey populations across Asia and selected global regions.

Region	Country/Area	Estimated Donkey Population	Year of Estimation	References
Global	Worldwide	Approximately 50 million	2023	[2,5]
Africa	Continental share	Approximately 30.5 million (≈61% of the globe’s donkey population)	2023	[2,4,5]
Asia (Total)	Continental share	Approximately 13 million (≈26% of the globe’s donkey population)	2023	[2,4,5]
South Asia	Pakistan	5.9 million	2023–2024	[36]
	India	1.2 million	2019	[37]
	Afghanistan	1.52 million	2023	[39,40]
East Asia	China	2.68 million	2020	[54,55]
Central Asia	Kazakhstan	0.030	2023	[39]
	Uzbekistan	0.088	2023	[39]
Western Asia	Iran	1.53	2023	[39,53]
Americas	Continental share	Approximately 6.5–6.6	2018–2023	[4,56,57]
The European continent and Oceania	Combined share	Approximately 0.13 million	2023	[4,57,58]

**Note:** Minor variations in population figures across countries (e.g., China: 2.60–2.68 million; Iran: 1.50–1.53 million) reflect differences in data sources, reporting years, and national estimation methods used in the referenced databases.

## Data Availability

All the data are available in the manuscript.
